# Measuring refugees’ capabilities: translation, adaptation, and valuation of the OxCAP-MH into Juba Arabic for use among South Sudanese male refugees in Uganda

**DOI:** 10.1186/s41687-024-00720-0

**Published:** 2024-04-02

**Authors:** C.F. van der Boor, D. Taban, K. Ismail, J. Simon, B. Roberts, D. Fuhr, W.A. Tol, G. Greco

**Affiliations:** 1 Faculty of Public Health and Policy, London School of Hygiene and Tropical Medicine, 15-17 Tavistock Place, London, WC1H 9SH UK; 2 HealthRight International, Plot 855, Mawanda Road -Kamwokya, Kampala, Uganda; 3 Department of Health Economics, Center for Public Health, Medical University of Vienna, Kinderspitalgasse 15, Vienna, 1090 Austria; 4 Department of Psychiatry, University of Oxford, Warneford Hospital, Oxford, OX3 7JX UK; 5 Department of Prevention and Evaluation, Leibniz Institute for Prevention Research and Epidemiology, Achterstraße, 30D-28359 Bremen, Germany; 6 Health Sciences, University of Bremen, Bremen, Germany; 7 Department of Public Health, University of Copenhagen, Bartholinsgade 4, bg. 9, 1356 København K, CSS, bg. 9, Building: 9.2.16, Copenhagen, Denmark; 8 Athena Research Institute, Vrije Universiteit Amsterdam, Amsterdam, The Netherlands

**Keywords:** Capability approach, Mental health, Uganda, PROMS, Refugees

## Abstract

**Background:**

Forcibly displaced populations are highly vulnerable to psychosocial distress and mental disorders, including alcohol misuse. In an ongoing trial that seeks to develop a transdiagnostic intervention addressing psychological distress and alcohol use disorders among conflict-affected populations, we will carry out a cost-effectiveness evaluation using a capability-based Oxford Capabilities Mental Health (OxCAP-MH) measure. The OxCAP-MH is a 16-item questionnaire developed from the Capability Approach, that covers multiple domains of functioning and welfare. The aim of the current paper is to present the results of the translation, cultural adaptation and valuation of the OxCAP-MH into Juba Arabic for South Sudanese refugees living in Uganda.

We adhered to the official Translation and Linguistic Validation process of the OxCAP-MH. To carry out the translation, the Concept Elaboration document, official English version of the OxCAP-MH, and the Back-Translation Review Template were used. Four independent translators were used for forward and back translations. The reconciled translated version was then piloted in two focus group discussions (N = 16) in Rhino refugee settlement. A most important to least important valuation of the sixteen capability domains covered in the OxCAP-MH was also done.

**Results:**

The Juba Arabic version of the OxCAP-MH was finalized following a systematic iterative process. The content of the Juba Arabic version remained unchanged, but key concepts were adapted to ensure cultural acceptability, feasibility, and comprehension of the measure in the local context of Rhino refugee settlement. Most participants had low levels of literacy and required support with filling in the tool. Participants suggested an additional capability that is currently not reflected in the OxCAP-MH, namely access to food. Furthermore, discussions around the valuation exercise of the sixteen domains led to two separate importance scales, which showed relevant differences.

**Conclusions:**

In this context, the OxCAP-MH was considered culturally acceptable. The valuation exercise proved cognitively demanding. Participants voiced confusion over how to answer the questions on the OxCAP-MH instrument due to low levels of literacy. These concerns invite consideration for future research to consider how measures such as the OxCAP-MH can be made more accessible to individuals with low literacy rates in resource poor settings.

## Background

Around the world, every two seconds, an individual is forcibly displaced due to conflict, violence, or persecution [1]. The significance of pre-migration traumatic events as predictors of mental health outcomes in conflict-affected communities is well-recognized [2, 3]. In addition, there is a growing awareness of the importance of ongoing post-migration stressors—such as unemployment and loss of social networks—for mental health, wellbeing, and quality of life. Multiple calls have been made for improved understanding of how mental health and psychosocial support (MHPSS) interventions can effectively cater to the specific needs of conflict affected populations in humanitarian settings [4, 5].

When evaluating psychological interventions, metrics for evaluating effectiveness and cost-effectiveness are often used. Conventional outcome measures such as quality-adjusted life years (QALYs) and disability-adjusted life years (DALYs), are used to estimate the cost-effectiveness of an intervention compared to a different one or compared to the status quo. This exercise is helpful to inform the most efficient allocation of scarce resources. QALYs quantify the combined quality of life and duration of lived years, while DALYs represent years lost due to ill-health, disability, or premature death [6]. Whilst QALYs and DALYs have been extensively utilized for the evaluation of healthcare interventions, it has been argued that they may not be sufficiently comprehensive to holistically evaluate interventions targeting a broader wellbeing perspective [7–9]. These metrics might fall short in encapsulating all critical outcomes beyond health that could influence an individual’s quality of life, leading to an underestimation of the full impact of the intervention [7]. For instance, the widely used EuroQoL 5 dimension and 5 level measure (EQ-5D-5L) [10], which is a measure that aims to describe and value health for economic evaluations, delineates health related quality of life across five dimensions: mobility, self-care, usual activities, pain/discomfort and anxiety/depression [10]. Concerns have arisen regarding its limitations in comprehensively capturing non-health dimensions of well-being that are important to an individuals’ mental health such as attachment, relationships, and enjoyment [7, 11, 12]. Given that MHPSS interventions in humanitarian settings can potentially manifest effects that extend beyond health-related quality of life outcomes, such as social integration, reduced stigma and mitigation of gender based violence, outcome measures need to include consideration for both health and beyond-health dimensions in such contexts [7, 13, 14].

Amartya Sen’s Capability Approach offers an alternative approach to measuring and valuing health intervention outcomes, potentially addressing the limitations of conventional cost-effectiveness measurement approaches [15–17]. The Capability Approach emphasizes the importance of meaningful choices and equitable opportunities in sustaining and enhancing health and quality of life across social, economic, and environmental dimensions [16]. Therefore, it provides a wider space to evaluate well-being. A recent review identified fourteen capability-based instruments for the economic evaluation of public health interventions [18], with the Oxford-Capability Questionnaire for Mental Health (OxCAP-MH) being the only capability-based wellbeing measure designed and validated for the area of mental health [19, 20].

The OxCAP-MH is a multidimensional sixteen item measure that was developed based on the list of ten central capabilities developed by Martha Nussbaum that she claimed sustain human life and dignity, namely life; bodily health; bodily integrity; senses, imagination and thought; emotions; practical reason; affiliation; other species; play; and control over one’s environment [21]. The OxCAP-MH has previously been translated and validated both in high-income [22, 23] and low-income settings [24] including Austria/Germany, Hungary and Uganda [22, 24, 25].

The objective of our study is to translate, adapt, and validate the OxCAP-MH for use among Juba Arabic speaking South Sudanese refugees in Uganda. Uganda is the largest host country for South Sudanese refugee, currently accommodating an estimated 931,666 individuals from South Sudan, primarily due to escalating violence that intensified in December 2013 [26]. The translation and validation process for the OxCAP-MH is a component of an ongoing trial evaluating the effectiveness and cost-effectiveness of a transdiagnostic intervention addressing mental distress and alcohol use disorders among conflict-affected populations (https://www.lshtm.ac.uk/change) [27]. The randomized controlled trial evaluates various outcomes, including percentage of abstinent days, depression, anxiety, post-traumatic stress disorder, intimate partner violence perpetration, and health-related quality of life through EQ-5D-5L and the OxCAP-MH.

## Methods

The translation and cultural validation of the English OxCAP-MH into Juba Arabic was coordinated by the CHANGE research team based at the HealthRight International office in Arua, northen Uganda, and at London School of Hygiene and Tropical Medicine (LSHTM).

**Fig. 1:**
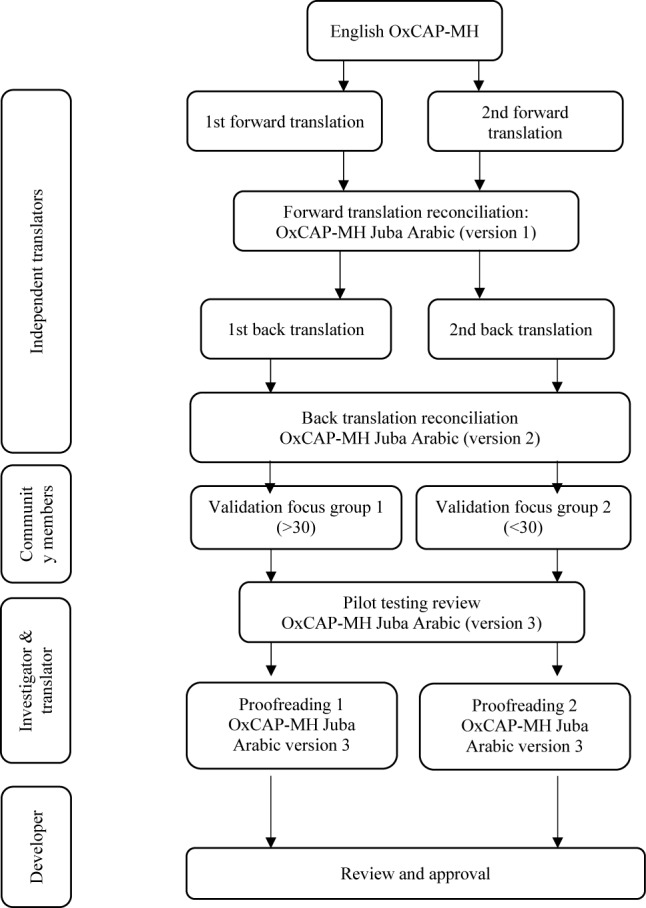
Translation and validation of the OxCAP-MH English version into Juba Arabic [adapted from 24]

The team in Arua consisted of four independent translators and a research fellow LSHTM. The official Translation and Linguistic Validation (TLV) process provided by the instrument’s guardian at the Medical University of Vienna was followed. This was designed using the international principles of good practice for the translation and cultural adaptation of patient-reported outcomes measures [28]. Beyond the TLV process, participants also carried out a least important to most important valuation of each of the sixteen capability domains covered in the English version of the OxCAP-MH measure by adapting a recent valuation study in Austria [29]. The steps of the translation and validation process followed in the current project are outlined in Fig. 1. The guardian of the tool (author JS) was involved in the conceptualization of the methodology and reviewed the overall translation process.

### Forward translation and forward translation reconciliation

Two initial forward translations were done from the English measure into Juba Arabic. Guided by the concept elaboration document provided by the authors of the OxCAP-MH, the forward translations were carried out by two independent translators based in Arua. Both translators are Ugandan nationals, native English and Juba Arabic speakers. They have extensive experience in carrying out translations of research tools and measures. Following the forward translations, a reconciliation exercise was done between them which resulted in a first Juba Arabic version (OxCAP-MH Juba Arabic V1).

### Back-translation and review

The OxCAP-MH Juba Arabic V1 was sent to two different independent Juba Arabic speakers for blinded back-translation, as specified in the concept elaboration document. One of the translators works as a research assistant at HealthRight International, and the other is a lay-healthcare worker. Both are fluent in Juba Arabic and English. The back translations were reconciled by the two translators and resulted in a new version of the OxCAP-MH (OxCAP-MH Juba Arabic V2).

### Pilot testing and validation workshop

#### Participants

We approached all people who had previously participated in the treatment cohort component of the CHANGE study telephonically. All sixteen agreed to participate and were divided into two workshops by age; South Sudanese adult males above 30 years old (N = 8), and South Sudanese adult males under 30 (N = 8). Thirteen participants had low literacy levels. For an overview of the sociodemographic information of participants see Table 1.

**Table 1:** Sociodemographic characteristics of participants

Age		36 (range 23–58)
Gender	Male	N = 16 (100%)
Ethnicity or tribe	Kakwa Other	N = 11 (69%) N = 5 (31%)
Time in settlement	6 years 5 years	N = 10 (63%) N = 6 (38%)
Highest level of education	Beyond Primary No schooling/primary school	N = 6 (37%) N = 10 (63%)
Marital status	Married Separated/never married	N = 10 (63%) N = 6 (37%)
More than one child	Yes No NA	N = 2 (13%) N = 12 (75%) N = 2 (13%)
Work status	Formal employment Farming Casual laborer Unemployed	N = 2 (13%) N = 8 (50%) N = 2 (13%) N = 4 (25%)
Literacy rate (as observed in the workshops)	High literacy Low literacy	N = 3 (19%) N = 13 (81%)

#### Procedures

Two validation workshops took place on 23 August 2022 in the Ofua 2 village within Rhino refugee settlement. The aim of the workshops was to review the OxCAP-MH Juba Arabic V2 with South Sudanese people living in the settlement, to determine its understandability and acceptability in this context. The first workshop was facilitated by the first translator involved in the forward translation, and the second by a translator involved in the back translation. The research fellow accompanied both workshops as she has expertise in the Capability Approach, and four extra research assistants were available.

Participants began by completing the OxCAP-MH Juba Arabic V2 on paper, with research assistants available for support. They then shared their impressions and reviewed each question individually. Participants were asked to raise their hand if a question was unclear; if not, they confirmed any suggested changes. These ‘hand raises’ were recorded (see Table 2). Next, participants discussed: (i) clarity of response options; (ii) any challenging words in the questions; (iii) potential alternative wording; and (iv) their own interpretation of each of the items. This sparked various discussions, especially regarding word changes. The facilitator, research fellow, and research assistants documented these talks and the final consensus.

**Table 2:** Changes that were suggested in the workshops on the OxCAP-MH Juba Arabic version 2 measure

Item	Content	Change requested by participants (Y/N)	Total number of respondents who suggested a change	Concern raised	Changes made
Q1	Does your health in any way limit your daily activities, compared to most people of your age?	Yes	9	The word chosen to translate ‘activities’ was not well understood	A synonym was chosen without changing the meaning of the question
Q2	Are you able to meet socially with friends or relatives?	Yes	6	There was a grammatical error	The error was rectified
Q3	In the past 4 weeks, how often have you lost sleep over worry?	Yes	11	The translation of ‘thoughts and worries’ were considered an outdated word	A more commonly used word was chosen
Q4	In the past 4 weeks, how often have you been able to enjoy your recreational activities?	Yes	5	The word chosen to translate ‘activities’ was not well understood	The same change was made as in Q1 to better reflect the word ‘activities’
Q5	How suitable or unsuitable is your accommodation for your current needs?	Yes	7	The translation for ‘suitable’ and ‘unsuitable’ was not clear	To make it more understandable this was changed to ‘good’ and ‘not good’
Q6	Please indicate how safe you feel walking alone in the area near your home	Yes	15	The translation for the word ‘safe’ was rectified as the translation used was urban centered	‘Safe’ was translated as ‘moving freely’, using a word that is understood across both urban and rural settings
Q7	Please indicate how likely you believe it to be that you will be assaulted in the future (including sexual and domestic assault)	No	0		
Q8	How likely do you think it is that you will experience discrimination? (Discrimination categories: Race/ethnicity, Gender, Religion, Sexual orientation, Age, Health or disability (incl. mental health), Other)	Yes	4	The translation given for Gender refers to sex rather than gender	The change was made to gender
Q9a	I am able to influence decisions affecting my local area	No	0		
Q9b	I am free to express my views, including political and religious views	No	0		
Q9c	I am able to appreciate and value plants, animals and the world of nature	No	0		
Q9d	I am able to respect, value and appreciate people around me	No	0		
Q9e	I find it easy to enjoy the love, care and support of my family and/or friends	No	0		
Q9f	I am free to decide for myself how to live my life	Yes	9	Free is difficult to translate, and should be translated to ‘able’	Instead of ‘I am free’ it was translated to ‘having freedoms’
Q9g	I am free to use my imagination and to express myself creatively (e.g. through art, literature, music, etc.)	Yes	4	There was a misunderstanding with the word ‘imagination’, an alternative was offered to make it more understandable and culturally relevant	Opted for the locally more appropriate translation of ‘thoughts’ instead of ‘imagination’
Q9h	I have access to interesting forms of activity (or employment)	Yes	17	Same issue as Q1	The same change was made as in Q1

#### Ethics

Workshops commenced with a verbal review of the participant information sheet and consent form, followed by an opportunity for questions. Participants with low literacy had the option to provide consent with a thumbprint, which was co-signed by a research assistant. The sessions were conducted in Juba Arabic and audio recorded. Participants received refreshments and a bar of soap as a token of appreciation for their time.

### Most important to least important valuation

Following the piloting of the OxCAP-MH Juba Arabic V2, a most important to least important valuation exercise of each of the sixteen capability domains of the OxCAP-MH was carried out. The sixteen dimensions were taken from Helter and colleagues [29] and translated into Juba Arabic prior to the workshops. Each of the sixteen dimensions were printed on a piece of paper, and participants were asked to rank the dimensions from least important (1) to most important (16). The ranking was visualized using raw beans where the number of beans represented the weighting given to each domain (i.e., if a domain was ranked as least important (1), one bean was placed). Participants were also asked if they thought any domains relevant to the local context were missing.

### Pilot and validation review

Following the pilot and validation, the written notes were reviewed by the in-country researchers and the research fellow. Suggested changes were made to increase the relevance and clarity of the measure, resulting in the OxCAP-MH Juba Arabic V3.

### Proof reading and review

A lay healthcare worker fluent in English and Juba Arabic who had not previously been involved in the translation process proof-read the translation. The third version was reviewed, and final grammatical edits were made in discussion with the in-country researcher. This version was reviewed and approved as the official Juba Arabic version of the OxCAP-MH by the developers.

## Results

### Pilot and validation

The OxCAP-MH Juba Arabic V3 is largely equivalent to the English OxCAP-MH measure [19] in terms of meaning and measurement. In line with the English version, it contains sixteen items. Given the high levels of illiteracy in the context, participants were not able to independently fill in the measure and required support herewith (i.e., interview format).

A formal and official TLV process (as required by the developers of the OxCAP-MH) was followed, which resulted in three versions of the Juba Arabic OxCAP-MH measure. The first version was developed following the reconciliation of the first two forward translations. The second version was created after the back translation reconciliation. The third version emerged after the pilot and validation workshops, and review thereof. Final grammatical edits were made during proofreading, resulting in the officially approved final version 3.

All participants who took part in the workshops were men, as the CHANGE project works exclusively with male refugees. The mean age across the two workshops was 36 years (range 23–58, see Table 1). Each of the two workshops lasted on average 2,5 hours which included the piloting and validation of the translated OxCAP-MH, and the least important to most important valuation.

At the start of the workshops, participants were asked to fill in the OxCAP-MH Juba Arabic version 3, with support from the research team. The first translation was read verbatim, and examples were provided if the translation was not understood on its own. Participants struggled with filling in the tool independently due to a combination of low literacy and not being accustomed to filling out self-report forms, including where to put the chosen response. Participants noted that:

**Participant 1:** For me the questionnaire is okay because what I did not understand properly, your colleague has helped me.

**Participant 3:** The questions are fine, if they read for me the questions slowly, I can understand them very well but if they are read at a high speed, I can easily not understand some of the statements.

For each question on the measure, participants were asked to raise their hand if they thought the question was clear. As shown in Table 2, translation wording changes were suggested for ten of the sixteen items (63%). The questions that were well translated and easily understood were questions related to being assaulted in the future (Q7), being able to influence local decisions (Q9a), freedom to express political and religious views (Q9b), appreciating and valuing plants, animals, and the world of nature (Q9c), being able to respect, value and appreciate others (Q9d), and enjoying the love, care and support of family and friends (Q9e). Most requested changes involved specific words, rather than underlying concepts more generally.

Feedback was also given regarding the translation of the response options. The only requested change was to the five-point response option of Q6 (‘very safe’, ‘fairly safe’, ‘neither safe nor unsafe’, ‘fairly unsafe’, ‘very unsafe’). Like the question itself, the word ‘safe’ was translated to being able to ‘move freely’, as this was a closer translation to the intended definition in English.

Q1 (‘Does your health in any way limit your daily activities, compared to most people of your age?’) was difficult to understand, particularly the translation of ‘daily activities’. This was translated in the form of ‘things you do all the time’. Similarly, in Q3 (‘In the past 4 weeks, how often have you lost sleep over worry?’), the translation provided for worry was considered outdated and was equated to ‘anger’, therefore this was changed to reflect a more up to date word for the final version.

**Moderator:** Can you please tell us how you understood the question?

**Respondent 6:** To me it is like how do you stay in the past 4 weeks; did you stay worried or not worried?

**Respondent 7:** I didn’t stay always *galag* (worried or angry) and so the answer is like sometimes, so in the past 4 weeks, there have been sometimes when I was *galag* because there was a time I went to my garden and I found the cows had eaten all my crops so then I was really *galag*.

**Respondent 2:** (laughing) that is anger not worry.

Throughout the workshops, participants drew on their day-to-day experiences to make sense of the questions and the wording:

**Moderator:** The next one reads *I am free to express my views, including political and religious views* (Q9b). Those who think they have understood this question should raise their hands.

**Participant:** Some of us are not understanding this question very well, the word *huur* (free) is confusing some us. But for me, whenever I go to church the priest always tells us that we should keep our hearts free from severe thoughts. So I think the word *huur* is simply meaning free or freedom here.

By the end of the workshop, changes were proposed to ten of the questions and one of the response options. These changes were agreed and incorporated into the OxCAP-MH Juba Arabic version 3.

### Most important to least important valuation

Following the individual item review, participants were invited to rank the sixteen capability dimensions that underly the OxCAP-MH. Both groups ranked the domain of ‘*I find it easy to enjoy the love care and support of my family and/or friends*’ in the top three most important capabilities. Furthermore, both groups ranked ‘*I am free to influence decisions affecting my local area’* in the 10th position, and ‘I am able to respect, value and appreciate people around me’ as the 11th most important. The rest of the domains were ranked differently across the two groups. The final ranking that was agreed in each group is shown in Table 3.

**Table 3:** Ranking of the OxCAP-MH dimensions from most important (16) to least important (1)

	Group 1	Group 2
16	I find it easy to enjoy the love, care and support of my family and/or friends	I am able to appreciate and value plants, animals and the world of nature
15	I am able to meet socially with friends or relatives	I have access to interesting forms of activity (or employment)
14	I am able to appreciate and value plants, animals, and the world of nature	I find it easy to enjoy the love care and support of my family and/or friends
13	My health does not limit my daily activities in any way compared to most people of my age	I am free to decide for myself how to live my life
12	I am free to express my views, including political and religious views	I am able to meet socially with friends or relatives
11	I am able to respect, value and appreciate people around me	I am able to respect, value and appreciate people around me
10	I am free to influence decisions affecting my local area	I am free to influence decisions affecting my local area
9	I have access to interesting forms of activity (or employment)	I do not experience discrimination
8	I am able to use my imagination and to express myself creatively (e.g. through art, literature, music, etc)	My accommodation is suitable for my needs
7	I am not assaulted (including sexual and domestic assault)	I am able to use my imagination and to express myself creatively (e.g. through art, literature, music, etc)
6	My accommodation is suitable for my needs	I am able to enjoy my recreational activities
5	I am able to enjoy my recreational activities	I am free to express my views, including political and religious views
4	I feel safe walking alone in the area near my home	My health does not limit my daily activites in any way compared to most people of my age
3	I am free to decide for myself how to live my life	I do not lose sleep over worry
2	I do not lose sleep over worry	I feel safe walking alone in the area near my house
1	I do not experience discrimination	I am not assaulted (incl. sexual and domestic assault)

Participants were invited to reflect on whether they considered any key domains to be missing. In the first focus groups, one additional domain was suggested: *access to food*.

**Respondent 1**: One important thing for our well-being is eating

**Interviewer:** What is it important about eating for us to ask?

**Respondent 1:** Food helps us; as we work hard on the farms to get food; it helps our body to function as well as our children need food to attend school.

**Interviewer:** What question should we ask about eating?

**Respondent 1:** Am able to get access to food through organizations.

**Respondent 2:** The thing is being able to grow food for ourselves, as the problem here is acquiring land to cultivate; we need to rent land and sign an agreement, and also the crops when the weather affects us like what happened, there is a problem, so that is the content I am bringing. So it is about how to have access to food amidst natural calamities like drought.

Beyond *access to food*, participants also highlighted how the lack of basic needs impacts on other capabilities through the example of education:

**Respondent:** (…) Regarding education, we see some learners drop out at times because of other needs, so the question would be, how would they be helped.

**Interviewer:** So, what are those other needs that are being prioritized over education?

**Respondent:** We have children who are orphans, we have children who are unaccompanied minors, we have girls who need material support (menstruation products), so, sometimes they cannot perform and drop out, so how can they be helped in that area.

**Interviewer:** And what is it about education that contributes to well-being?

**Respondent:** The materials be provided to them, maybe when they grow to our level they would have no problems, they will care for themselves.

These quotes highlight the lack of basic needs and high levels of insecurity that refugees in Rhino refugee settlement face including poverty, lack of material support, dependence on external support organizations, and climatic hazards including droughts that affect individuals’ abilities to grow crops.

## Discussion

Our study describes the cultural and linguistic validation and adaptation of the official Juba Arabic version of the OxCAP-MH [19] which was developed following a systematic iterative process in a humanitarian setting in Uganda. The Juba Arabic translation and adaptation was carried out together with a group of sixteen South Sudanese men living within Rhino refugee settlement. It is the first translation and adaptation of the OxCAP-MH for use in a humanitarian setting.

The current study methodology was rigorous and followed the principles of good practice for translation of patient-reported outcome measures developed by the International Society for Pharmacoeconomics and Outcomes Research’s (ISPOR) standards [28]. In the Juba Arabic version, wording changes were suggested for 63% of the items during the pilot testing and validation workshops. Most of the requested changes were grammatical in nature and pertained to different ways of expressing similar words in Juba Arabic, reflecting variations between, for instance, rural and urban linguistic forms. Six questions were well understood by the participants: Q7 (future assault), Q9a (influencing local decisions), Q9b (freedom of expression), Q9c (appreciating nature), Q9d (respecting others), and Q9e (enjoying support from others). This finding contrasts with the results of the translation and adaptation of the OxCAP-MH into Luganda in a distinct setting in Uganda [24]. During the translation into Luganda, the authors observed that significant cultural differences emerged for Q9b (freedom of expression), Q9c (appreciating nature), Q9g (imagination) and Q9h (access). The authors observed that these four questions introduced concepts that do not have cultural equivalents in Uganda. While the present study was also conducted in Uganda, the participants were South Sudanese, and thus, this discrepancy in findings related to concepts might stem from cultural distinctions among the different cultural groups involved. Conversely, in our study, participants encountered difficulties in comprehending the translated terms for ‘daily activities’ in Q1 and ‘worry’ in Q3. They highlighted that the provided translations for these terms were outdated, potentially indicating some geographical variances in the Juba Arabic used by the participants themselves and the initial translators of the tools. This discrepancy underscores the significance of conducting a comprehensive methodological process for translation and validation for assessment instruments to consider cultural and linguistic nuances. It has previously been reported that modifications tailored to local contexts and target populations are more likely to accurately evaluate the specific constructs of interest while upholding cultural sensitivity, unlike universal measures lacking cultural adaptation [30–32].

The ranking of the sixteen capability domains, from most important to least important, revealed significant differences between the two focus groups. Within Group 1 (respondents aged > 30), the capability domain regarded as the least important was ‘I do not experience discrimination’, whereas in Group 2 (respondents aged < 30), not being subjected to assault (including sexual and domestic assault) was identified as the least important capability. Additional investigation is needed to fully understand why these domains were considered least important. One possible reason could be that the study focused more on men’s perspectives, which might have led to some participants feeling uncomfortable or worried about discussing their fears of assault openly in a group setting. Earlier research has highlighted high rates of sexual violence affecting both female and male South Sudanese refugees in northern Uganda. One cross-sectional survey found that 30.4% of men reported having experienced sexual violence themselves or witnessed it against other men [33]. More recently, a study involving 447 male refugees residing in a Ugandan settlement reported that 13.4% had encountered sexual violence in the past year alone [34]. While the domain related to assault covers various forms of assaults, societal expectations and norms around masculinity might discourage men from openly discussing concerns about assault, particularly in a group setting. This potential reluctance could stem from the domain’s explicit inclusion and specification of sexual and domestic assault, however further research is needed to determine this.

With regard to the capability domains ranked as most important, the importance of the domain ‘I find it easy to enjoy the love, care and support of my family and/or friends’ has been replicated in other studies, whereby social support is identified as a key predictor of well-being and quality of life for conflict affected populations [35], including in Uganda [36]. The ranking of ‘I am able to appreciate and value plants, animals and the world of nature’ as most important capability domain in group 2 could be linked to the importance of farming within Rhino refugee settlement, given that it is one of the limited ways individuals can work and/or grow their own food. In 2019, the UNHCR interviewed 125 households and reported that 74% reported using their shelter plot for land cultivation to have more food, whilst of those 82% said this was insufficient to provide food for the household in the most recent harvest [37]. More recently, the Refugee Livelihoods and Resilience Sector Strategy for Uganda reported that agriculture employs 73% of refugees in Uganda [38]. In a recent qualitative exploration of psychological basic needs and wellbeing amongst refugees in Rhino settlement, participants reported that food and farming insecurity is highly linked to the basic psychological need for autonomy [39]. Respondents reported having limited access to food sources [39], which is also reflected in our study by the suggestion to add *access to food* as a central capability domain. Although food is typically categorised as a physical basic need, the challenge in the current context is the limited agency individuals have with regards to accessing and/or cultivating food in the context of high costs to rent plot, and climate change. As a result, access to food is almost entirely dependent on food aid, thereby severely limiting individuals’ choices and agency in terms of food acquisition.

The research methodology employed revealed several challenges inherent to conducting research within a resource-poor setting. Firstly, there was a very low level of literacy amongst participants in the focus group (only 19% were literate). These statistics mirror the reality within Rhino refugee settlement. An interagency livelihoods assessment conducted in 2017 by the UNHCR and World Vision reported that among the 785 surveyed refugees, merely 44% had achieved primary education, 25% had reached secondary education, and 25% remained either illiterate or semi-literate [40]. Moreover, participants who were literate expressed difficulties in comprehending how to complete the assessment tool due to a lack of familiarity with self-reported outcome measures. The current study underscored that the format of the OxCAP-MH, in line with most self-reported outcome measures, is tailored to a population with at least basic literacy skills, and some experience of filling in self-report measures. Although research staff can help administer the OxCAP-MH, this approach demands substantial labour and financial resources, and additionally carries potential biases [41–43]. Consequently, consideration needs to be given to how self-report measures might be made more inclusive, with the aim of mitigating quality-related risks and preventing the perpetuation of mental health disparities particularly in humanitarian settings.

Beyond these challenges, some further limitations need to be considered. During the translation and adaptation process, local researchers and research participants carried out the work, rather than using an official translation company (i.e., trained translators). While this approach carries inherent risks, it also presented an opportunity to tailor the tool to the real-world context of men residing in the Rhino refugee settlement. Secondly, we did not involve any South Sudanese women in the research process. Consequently, the finalized tool requires further piloting with South Sudanese women to validate its applicability within this demographic. Moreover, all the men who took part in the piloting and workshops were individuals previously engaged in the formative research of the CHANGE project. Consequently, they had recently undergone a brief psychological intervention aimed at improving their mental health wellbeing. While this circumstance is unlikely to impact the translation of the OxCAP-MH tool, it might have influenced the valuation exercise of capability domains.

## Conclusions

In this study we developed the official Juba Arabic version of the OxCAP-MH measure. This measure is both culturally and linguistically appropriate for use with South Sudanese refugee men living in Uganda and is feasible for the measurement of capability based mental health outcomes. In the pilot and validation exercise, participants confirmed that the questions on the OxCAP-MH represent relevant aspects of their quality of life and wellbeing in the refugee settlement. However, it was also noted that due to low levels of literacy participants found it difficult to understand the self-reporting format of the measure. These concerns invite consideration for future research to consider how measures such as the OxCAP-MH can be made more accessible to individuals with low literacy rates in resource poor settings. Furthermore, the ranking of the sixteen capability domains, from most important to least important, revealed significant differences between the two focus groups and more research is required to disentangle these differences. The developed Juba Arabic version of the OxCAP-MH can be used as an alternative or in addition to other health related quality of life outcomes for economic evaluation of psychological interventions.

## Data Availability

Participants of this study did not agree for their data to be shared publicly, therefore supporting data is not available.

## List of abbreviations

DALY: Disability-adjusted life years
EQ-5D-5L: EuroQoL 5 dimension and 5 level measure
LSHTM: London School of Hygiene and Tropical Medicine
MHPSS: Mental health and psychosocial support
OxCAP-MH: Oxford Capabilities Mental Health measure
PM+: Problem Management Plus
QALY: Quality-adjusted life year
TLV: Translation and Linguistic Validation
